# IFN-τ Mediated Control of Bovine Major Histocompatibility Complex Class I Expression and Function *via* the Regulation of bta-miR-148b/152 in Bovine Endometrial Epithelial Cells

**DOI:** 10.3389/fimmu.2018.00167

**Published:** 2018-02-02

**Authors:** Haichong Wu, Kangfeng Jiang, Shuai Guo, Jing Yang, Gan Zhao, Changwei Qiu, Ganzhen Deng

**Affiliations:** ^1^Department of Clinical Veterinary Medicine, College of Veterinary Medicine, Huazhong Agricultural University, Wuhan, China

**Keywords:** IFN-τ, major histocompatibility complex class I, TLR4, bta-miR-148b/152, bovine endometrial epithelial cells

## Abstract

IFN-τ, a type I interferon produced by the trophoblasts of ruminants, has various important immune functions, including effects on the expression of major histocompatibility complex (MHC) class I (MHC-I). A previous study has reported that IFN-τ promotes the expression of MHC-I molecules on endometrial cells. However, the immunological mechanisms by which IFN-τ regulates MHC-I molecules remain unknown. Here, we investigated which microRNA (miRNAs) may be involved in the regulation of MHC-I molecule expression and function in bovine endometrial epithelial cells (bEECs). By using TargetScan 6.2 and http://www.microRNA.org, two miRNAs were suggested to target the 3′UTR of the bovine MHC-I heavy chain: bta-miR-148b and bta-miR-152. Dual luciferase reporter and miRNA mimic/inhibitor assays suggested that bta-miR-148b/152 were negatively correlated with bovine MHC-I heavy chain genes. The function of the MHC-I heavy chain was then investigated using qRT-PCR, ELISA, western blotting, immunofluorescence, and RNA interference assays in primary bEECs and an endometrial epithelial cell line (BEND). The results demonstrated that bta-miR-148b/152 could promote TLR4-triggered inflammatory responses by targeting the bovine MHC-I heavy chain, and the MHC-I molecule negatively regulated TLR4-induced inflammatory reactions may through the Fps-SHP-2 pathway. Our discovery offers novel insight into negative regulation of the TLR4 pathway and elucidates the mechanism by which bovine MHC-I molecules control congenital inflammatory reactions.

## Introduction

IFN, a type I interferon, is secreted by ruminant animal trophoblasts (1). It is responsible for maternal recognition of pregnancy in ruminants, which links the fields of developmental biology and reproductive immunology (2). Many experiments have shown that IFN-τ also has multiple biological functions, such as anti-proliferative, anti-inflammatory, and antiviral effects (3–5). The bovine major histocompatibility complex (MHC), also called the bovine leukocyte antigen (BoLA), is involved in the regulation of the immune response through antigen recognition by T lymphocytes (6). The MHC class I (MHC-I) region in mammals contains both classical and non-classical MHC-I genes (7). Classical MHC-I molecules, such as the MHC-I heavy chain, play important roles in the immune response (8, 9). Several studies have shown that IFN-τ promotes MHC-I expression on endometrial cells (10). However, how the immunological mechanisms of IFN-τ regulate MHC-I molecules remains unknown. Thus, the high-throughput sequencing technology was employed to investigate the specific microRNA (miRNA) library of IFN-τ on bovine endometrial epithelial cells (bEECs) cells (11).

MicroRNAs, which are endogenous RNAs of approximately 22 nucleotides, play major roles in the regulation of plants and animals *via* targeting mRNAs for disruption or translational repression (12). Previous research has indicated that miRNA plays an essential role in various physiological and pathological processes, including development, proliferation, and differentiation (13). There is some evidence to suggest that miR-148/152 specifically mediates HLA-G expression and other immune responses (14, 15). However, it is unknown whether any other miRNAs are involved in the regulation of bovine MHC-I molecules. Therefore, the primary purpose of this research was to identify candidate miRNAs that may regulate bovine MHC-I molecule expression and function.

Toll-like receptor (TLR) is a type I transmembrane protein with ectodomains containing leucine-rich repeats that recognizes different pathogen-associated molecular patterns and plays vital roles in host defenses against invading microbial pathogens (16, 17). TLR4 was the first identified mammalian TLR (18). It regulates immunologic responses against host molecules responsible for tissue injury and chronic inflammation (19). TLR4 is an important receptor for bacterial lipopolysaccharide (LPS) (20). After the binding of LPS, which triggers the activation of the myeloid differentiation factor 88 (MyD88)-dependent pathway, the nuclear transcription factor (NF)-κB stimulates inflammatory cytokine secretion (21, 22). A previous study showed that MHC-I molecules interact with TLR ligands and then suppress innate inflammatory responses (23).

Considering that miRNA is a highly conserved regulatory molecule in many different species, it is possible that the bovine MHC-I heavy chain is regulated by miR-148/152. By using TargetScan 6.2 and http://www.microRNA.org, two miRNAs were suggested to target the 3′UTR of the bovine MHC-I heavy chain: bta-miR-148b and bta-miR-152. In the current research, we investigated which one(s) of these miRNAs may be involved in the regulation of MHC-I molecule expression and function in bEECs. We demonstrated that down-regulated bta-miR-148b/152 could suppress LPS-triggered inflammatory responses by targeting the bovine MHC-I heavy chain, and the MHC-I molecule negatively regulated TLR4-induced inflammatory reactions may through the Fps-SHP-2 pathway. Therefore, bta-miR-148b/152 may act in the fine tuning of TLR4-triggered innate inflammatory responses.

## Materials and Methods

### Reagents

Recombinant bovine interferon-tau (IFN-τ, HPLC >97%) was purchased from Creative Bioarray (NY, USA). The commercial bovine endometrial cell line (BEND) was obtained from American Type Culture Collection (USA). The Anti-HLA Class I antibody (W6/32) was obtained from abcam (ab23755, UK). BEND cells were cultured and propagated as described in the instructions provided by ATCC.

### Cell Culture, Identification, and Treatment

Primary bEECs were detached and cultivated as described previously (24). Briefly, the uterus of Holstein cow was collected from a local slaughterhouse and immediately taken back to the laboratory in phosphate-buffered solution (PBS). Then, the uterus was cut up, digested with collagenase-I for 2 h, neutralized, and placed in a Petri dish. Cells were cultivated in DMEM/F12 containing 10% FBS and incubated with 5% CO_2_ at 37°C.

The cells were passaged in 12-well plates with cover slips, and we analyzed the expression of the epithelial-specific marker cytokeratin 18. Cells were fixed in formalin for 10 min and then washed three times with PBS. The cells were blocked with 10% normal goat serum (Invitrogen, USA) at room temperature for 30 min and then incubated with a primary cytokeratin 18 antibody (diluted 1:300 in PBS) overnight at 4°C. The secondary fluorescently labeled antibody, Dylight 594 antibodies (Bioss, China), was incubated with the cells for 45 min at room temperature. DAPI was used to stain the cell nuclei. Fluorescence images were obtained using confocal microscopy (Leica, Germany).

The bEECs were plated at 1 × 10^6^/mL in six-well plates and then challenged with IFN-τ (200 ng/mL) for 6 (TS group) or 12 h (TT group); untreated cells were used as control groups at the corresponding time points of 6 and 12 h (CS and CT groups, respectively).

### qRT-PCR Analysis

Total RNA was isolated by TRIzol agent following the producer’s directions (Invitrogen, USA). PCR was carried out with a SYBR^®^ Select Master Mix kit and PCR amplification system. The primers are listed in Table 1. The expression levels of related genes were standardized to β-actin expression using the 2^−ΔΔCt^ comparative method. For miRNA qRT-PCR, a commercial Hairpin-it™ miRNAs qPCR Quantification kit (GenePharma, China) was used. Specific primers for bta-miR-148b, bta-miR-152, and U6 were designed by GenePharma. qRT-PCR was carried out using a SYBR^®^ Select Master Mix kit and PCR amplification system. The expression of miRNAs was standardized to U6 expression using the 2^−ΔΔCt^ comparative method.

**Table 1:** Primers used for qPCR.

Name	Sequence (5′ → 3′): forward and reverse	Accession no.	Product size (bp)
MHC class I heavy chain	TATGTGGACGACACGCAGT	NM_001038518.2	188
	TCGCTCTGGTTGTAGTAGCC		
β-Actin	CTCTTCCAGCCTTCCTTCCT	BC102948	124
	GGGCAGTGATCTCTTTCTGC		

### Transfection with miRNA Mimics and Inhibitors

The bEECs were transfected with 50 nM bta-miR-148b and bta-miR-152 mimics, inhibitors, or corresponding negative control constructs (GenePharma, China) using Lipofectamine 2000 following the producer’s directions. Transfected cells were harvested at 24 h.

### Cytokine Assay

The concentrations of TNF-α and IL-1β in the supernatant of cells were detected with ELISA kits according to the producer’s directions. The optical density value was acquired at 450 nm with a microplate reader.

### Plasmid Construction

The predicted target sites of the 3′-UTR of the bovine MHC-I heavy chain gene or sequences containing mutated versions of the predicted target sites were cloned into the SacI and XhoI sites of the pGLO promoter vector (Promega, USA). The bta-miR-148b/152 target site 5′-ATGCACTGA-3′ in the bovine MHC-I heavy chain 3′-UTR was mutated to 5′-GTAGTATCC-3′ by site-directed mutagenesis. To prepare bta-miR-148b/152-GLO vectors, PCR amplification was performed on bta-miR-148b and bta-miR-152 sponges. The PCR products were digested with SacI and XhoI and cloned into the pmirGLO vector. The primers were as follows: bta-miR-148b sponge sense 5′-CACAAAGTTCTGTGATGCACTGAATCGACAAAGTTCTGTGATGCAC TGAC-3′ and antisense 5′-TCGAGTCAGTGCATCACAGAACTTTGTCGATTCAG TGCATCACAGAACTTTGTGAGCT-3′; bta-miR-152 sponge sense 5′-CCCCGTTC TGTCATGCACTGAATCGCCCAAGTTCTGTCATGCACTGAC-3′, and antisense 5′-TCGAGTCAGTGCATGACAGAACTTGGGCGATTCAGTGCATGACAGAACTTGGGGAGCT-3′.

### Luciferase Activity Analysis

HEK-293 cells were cultivated in DMEM containing 10% FBS in 24-well dishes with 5% CO_2_ at 37°C. Cells were transfected at 80% confluency with 2 µL of lipofectamine 2000 (Invitrogen, USA) in 100 µL of Opti-MEM I without serum, following the lipofectamine producer’s directions. Each plate was transfected with 80 ng of pmirGLO-MHC-I heavy chain/WT vector or pmirGLO-MHC-I heavy chain/MUT vector containing firefly luciferase and the pRLTK vector (Promega, USA) containing bta-miR-148b-GLO, bta-miR-152-GLO, or control-GLO (pmirGLO, Promega). Cells were then cultured for 24 h without changing the medium and were lysed for 15 min with 30 µL of Passive Lysis Buffer (Promega, USA) on a rocking platform. Firefly luminescence was normalized to renilla luminescence to calculate the relative luciferase activity using the Dual Luciferase Reporter Assay following the producer’s directions.

### Western Blot Analysis

Cells were lysed with lysis buffer containing phosphatase inhibitor and centrifuged at 10,000 *g* for 10 min. The protein concentration was determined with a BCA kit. Then, the same amount of protein from each sample was separated by SDS-PAGE and transferred onto a PVDF membrane. The membrane was incubated with specific antibodies at 4°C for 12 h after being placed in blocking buffer. Then, the membrane was incubated with secondary antibodies for 1 h at room temperature. The membrane was washed with PBS containing 0.05% Tween-20 three times, and the expression levels of proteins were then determined with a chemiluminescence system.

### RNA Interference Analysis

The sequences of small interfering RNA (siRNA) oligonucleotides targeting the MHC-I heavy chain transcript were as follows: siRNA MHC-I hc sense 5′-GGUCUUCGACCUCUUCCAUTT-3′ and antisense: 5′-AUGGAAGAGGUCGA AGACCTT-3′. Scrambled siRNA (sense: 5′-UUCUCCGAACGUGUCACGUTT-3′ and antisense: 5′-ACGUGACACGUUCGGAGAATT-3′) was used as a negative control. The siRNA was combined with lipofectamine 2000 to form complexes before transfection into each well. The complexes were added directly to the bovine endometrial epithelial cell line (BEND), and transfection was performed when cells were attached to the well. A total of 1 × 10^5^ cells/well were transfected with either siRNA MHC-I hc or negative control siRNA (GenePharma, China) in 24-well culture plates in a volume of 0.5 mL of serum-free DMEM. After the cells were cultivated for 24 h, the culture solution was replaced with the one used before transfection. After 48 h, the cells were harvested for RNA and protein extraction.

### Immunofluorescence Staining Analysis

The cells were fixed with 4% formalin for 10 min and then incubated with PBS containing 0.3% Triton X-100 (Sigma, USA) and 10% bovine serum albumin to permeabilize the cells and block interactions with nonspecific proteins. Next, the cells were incubated with specific antibodies against TLR4 or p65 for 12 h and then with Cy3 secondary antibodies in the dark for 2 h at room temperature. Finally, TLR4 or p65 protein was detected and immobilized using mounting media containing DAPI. The cell slices were viewed under a fluorescence microscope.

### Statistical Analysis

SPSS software was used for statistical analyses. Data are presented as the mean ± SEM of three independent trials. The data were analyzed with one-way ANOVA or Student’s *t*-test. *P* ≤ 0.05 indicated statistically significant differences.

## Results

### Cell Identification and the Expression of the MHC-I Heavy Chain and miRNAs in IFN-τ-Stimulated Cells

**Figure 1:**
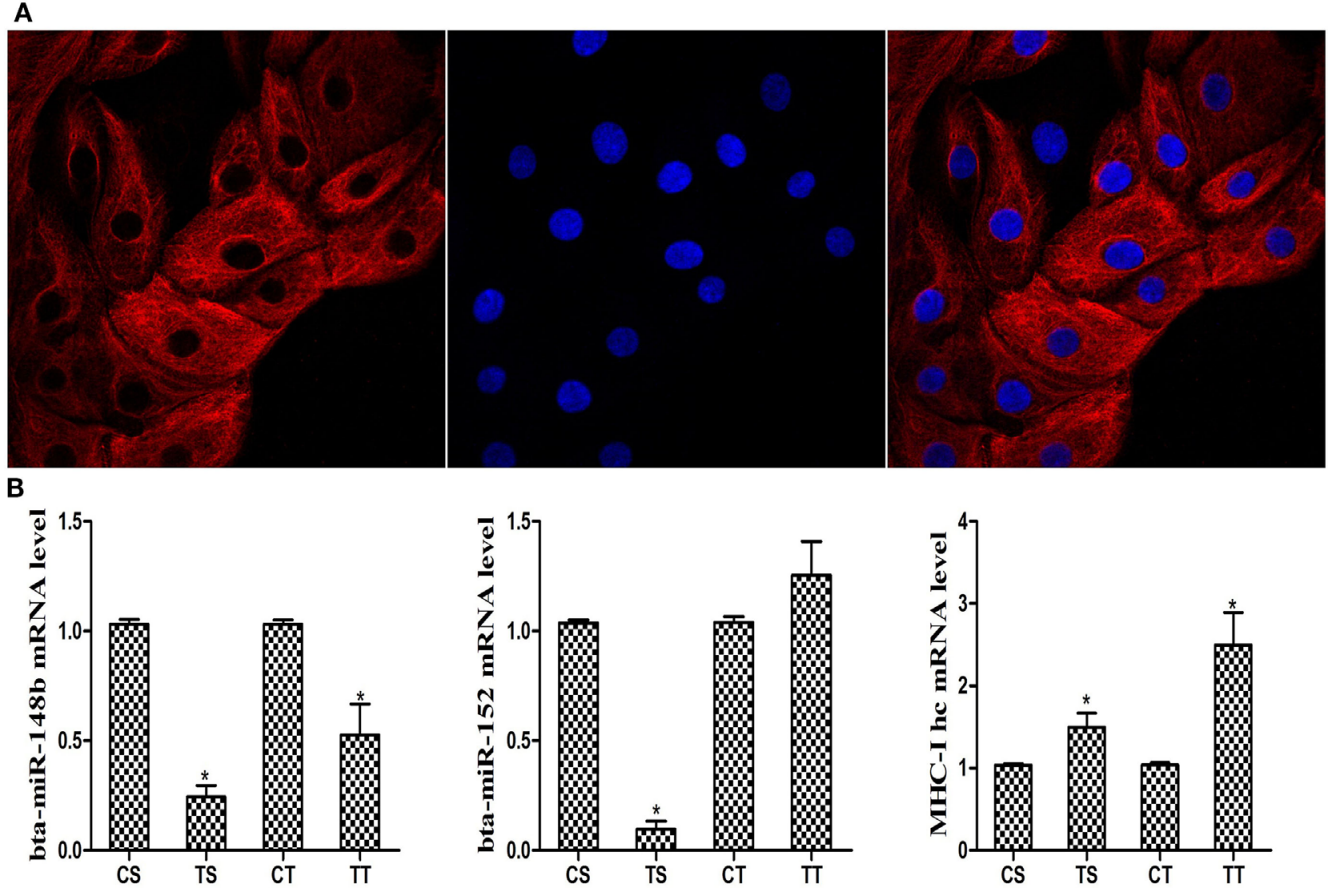
Cell identification and expression of the MHC class I (MHC-I) heavy chain and bta-miR-148b/152 in IFN-τ stimulated cells. **(A)** Bovine endometrial epithelial cells were pretreated with fluorochrome to observe endometrial epithelial cell integrity. The cell nucleus was marked with blue fluorescence. Cytokeratin 18 was labeled with a red fluorochrome (magnification 400×). **(B)** Relative bta-miR-148b/152 and MHC-I heavy chain mRNA expression levels in IFN-τ-stimulated cells were determined by qRT-PCR analysis. U6 was used as an internal control. The TS and TT groups were challenged with IFN-τ for 6 or 12 h. The CS and CT groups were untreated cells and were used as control groups at the corresponding time points of 6 and 12 h. Data are presented as the mean ± SEM of three independent experiments. *P < 0.05 versus the control group.

Cytokeratin 18 is an epithelial-specific marker that identifies bEEC integrity. bEECs were pretreated with DAPI to identify the nucleus. The results are shown in Figure 1A. Then, we analyzed the expression of bta-miR-148b and bta-miR-152 in bEECs challenged with IFN-τ using qRT-PCR analysis. The results showed that bta-miR-148b and bta-miR-152 expression was reduced, but the level of bovine MHC-I heavy chain mRNA was increased by IFN-τ treatment (Figure 1B).

### bta-miR-148b and bta-miR-152 Target the Bovine MHC-I Heavy Chain Gene

**Figure 2:**
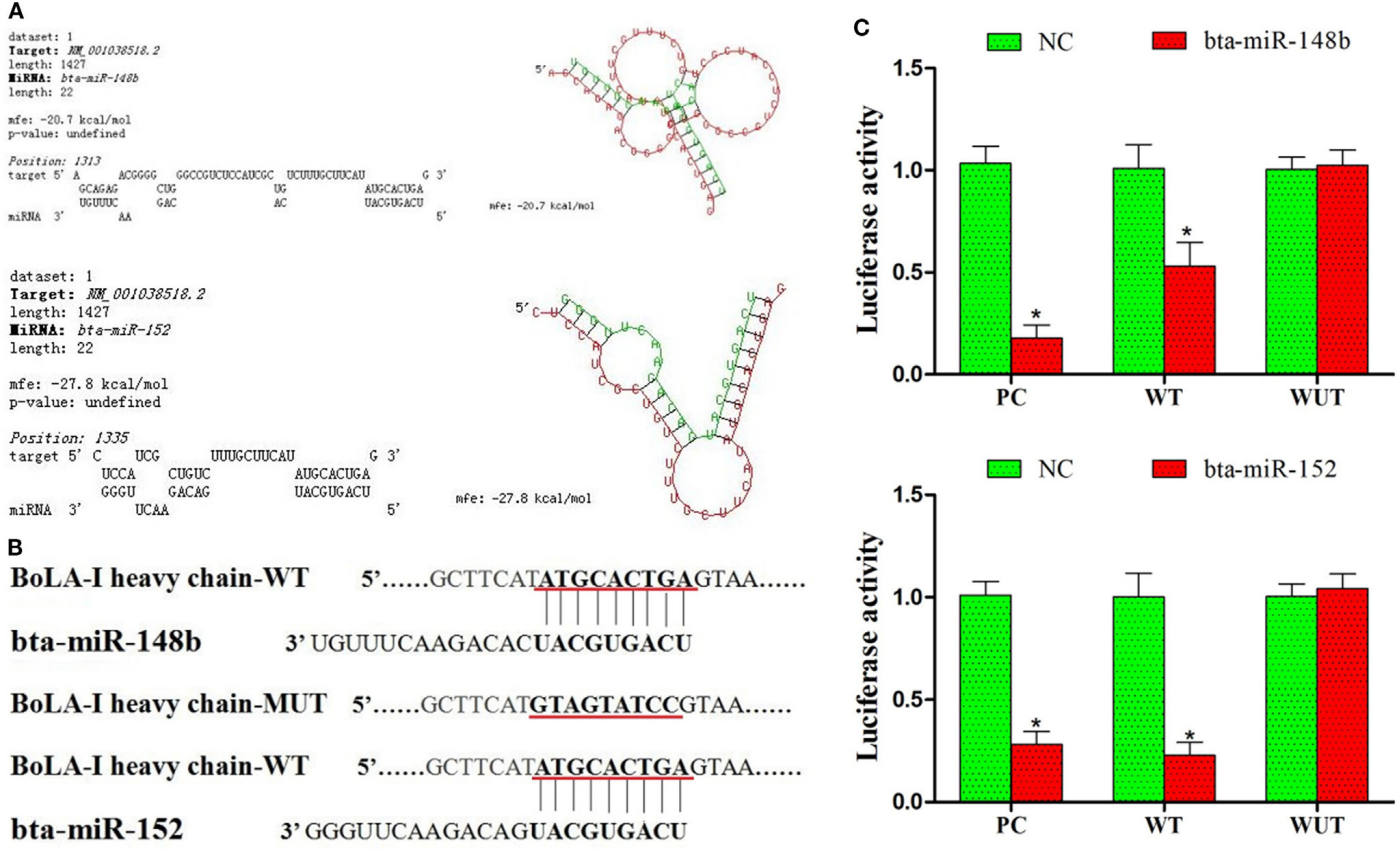
bta-miR-148b and bta-miR-152 target the bovine MHC class I (MHC-I) heavy chain gene. **(A)** Alignment of the 3′UTR of the bovine MHC-I heavy chain with the predicted microRNA (miRNAs). **(B)** The miR-148b/152 sequences are predicted to interact with MHC-I heavy chain 3′UTR were displayed. **(C)** Luciferase activity in bovine endometrial epithelial cells transfected with control miRNA, bta-miR-148b, or bta-miR-152. Luciferase activity was normalized by the ratio of firefly and Renilla luciferase signals. PC, positive control (bta-miR-148b sponge and bta-miR-152 sponge). WT, wild-type. MUT, mutant. Data are presented as the mean ± SEM of three independent experiments. *P < 0.05 compared to the negative control.

Additional assays were performed to determine whether bta-miR-148b and bta-miR-152 expression is involved in the regulation of bovine MHC-I molecules. Using the prediction software TargetScan6.2, we found that bta-miR-148b and bta-miR-152 could potentially target the 3′UTR of the MHC-I heavy chain according to the calculation of minimum free energy (Figure 2A). The miR-148b/152 sequences are predicted to interact with MHC-I 3′UTR were displayed in Figure 2B. To confirm that bta-miR-148b and bta-miR-152 directly bind the 3′UTR of the bovine MHC-I heavy chain, a dual luciferase reporter assay was performed in HEK-293 cells. We integrated a fragment of the bovine *MHC-I heavy chain* 3′-UTR containing the target sequence or a fragment in which the target sites were mutated into the luciferase reporter vector and cotransfected this vector with bta-miR-148b mimics, bta-miR-152 mimics, or scrambled oligonucleotides. We found that bta-miR-148b/152 mimics significantly reduced the luciferase activity of wild-type *MHC-I heavy chain* luciferase (*MHC-I heavy chain*-Luc) but had no effect on mutant *MHC-I heavy chain*-Luc (Figure 2C). This results suggested that bta-miR-148b/152 targeted the MHC-I heavy chain gene.

### bta-miR-148b/152 Inversely Correlates with the MHC-I Heavy Chain in bEECs

**Figure 3:**
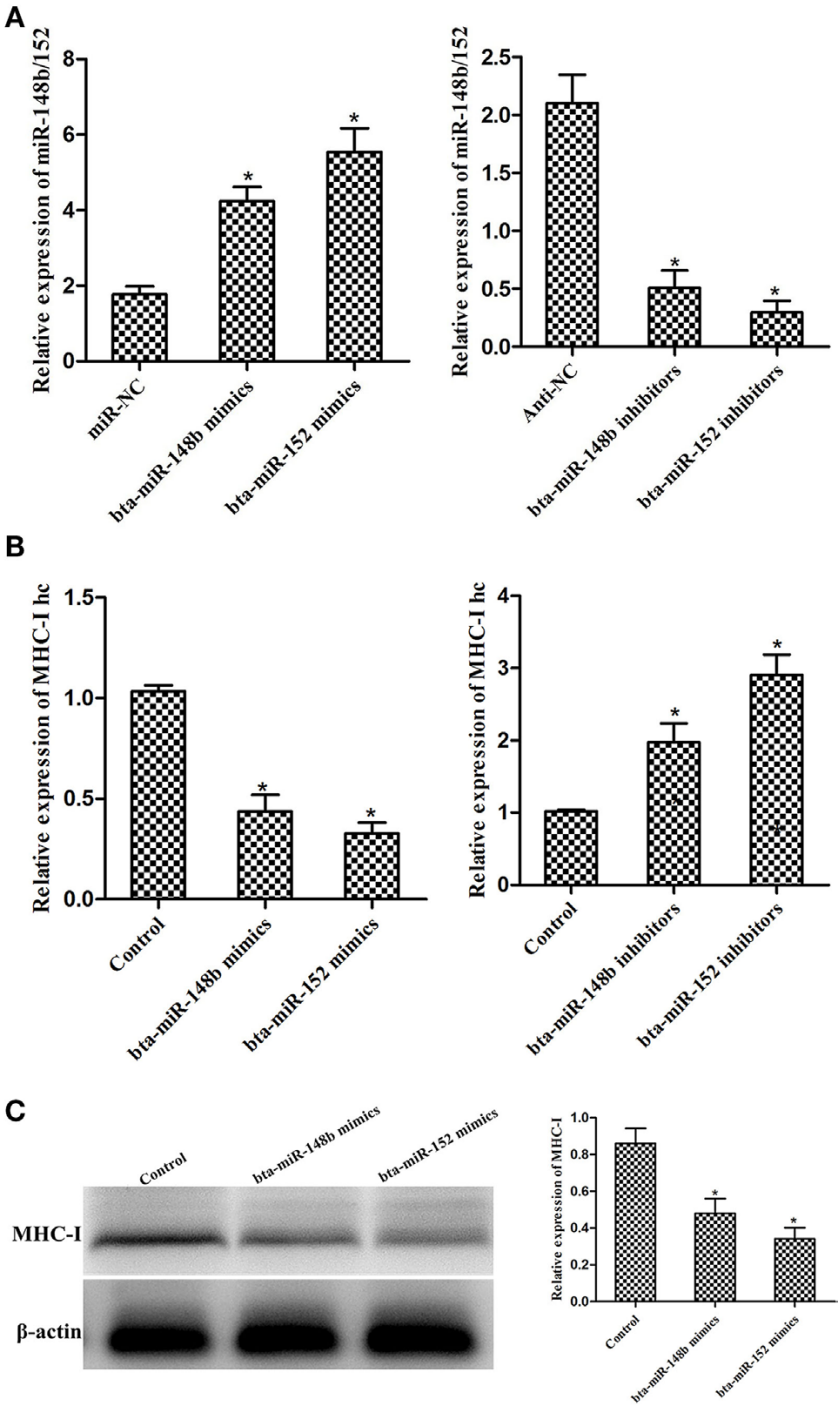
bta-miR-148b/152 inversely correlate with MHC class I (MHC-I) heavy chain in bovine endometrial epithelial cells (bEECs). **(A)** qRT-PCR analysis of bta-miR-148b/152 expression in bEECs after transfection with bta-miR-148b/152 mimics, inhibitors, or negative controls. U6 was used as an internal control. **(B)** qRT-PCR analysis of MHC-I heavy chain mRNA expression in bEECs after treatment with bta-miR-148b/152 mimics, inhibitors, or negative controls. **(C)** Western blot analysis of MHC-I expression in bEECs after treatment with bta-miR-148b/152 mimics or negative controls. β-actin was used as an internal control. Data are presented as the mean ± SEM of three independent experiments. *P < 0.05 compared to the negative control.

We observed that the expression of bta-miR-148b/152 increased after treatment with bta-miR-148b/152 mimics and decreased after treatment with bta-miR-148b/152 inhibitors (Figure 3A). To investigate whether bta-miR-148b/152 affect the regulation of the MHC-I heavy chain in bEECs, we measured the relative expression of the MHC-I heavy chain using immunoblotting and qRT-PCR methods. The results showed that MHC-I heavy chain expression was significantly decreased by bta-miR-148b/152 mimics but was increased by bta-miR-148b/152 inhibitors (Figures 3B,C). Thus, the data above indicated that bta-miR-148b/152 negatively affected MHC-I heavy chain expression.

### bta-miR-148b/152 Impact the Regulation of MHC-I Molecules in TLR4-Triggered Inflammatory Responses

**Figure 4:**
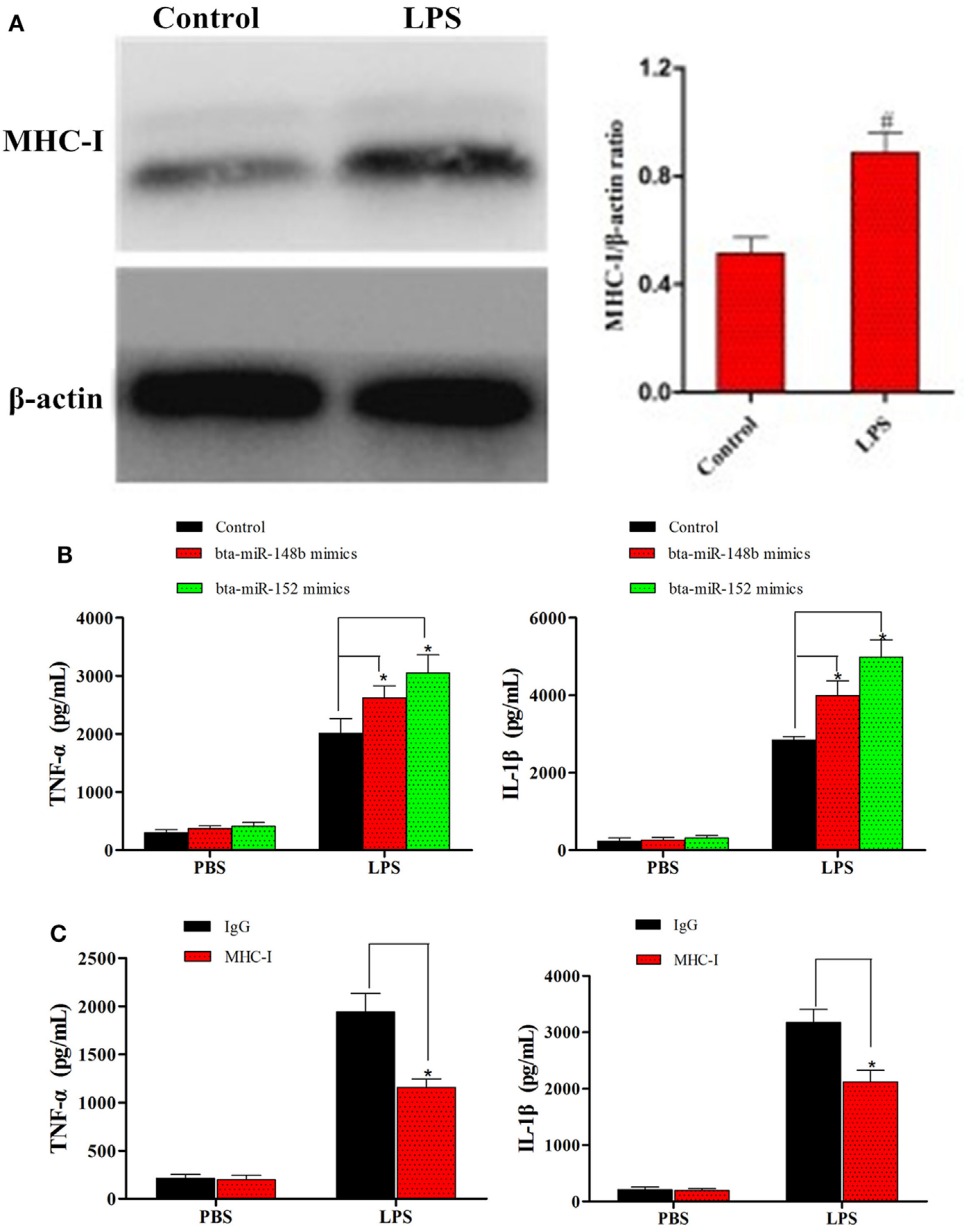
bta-miR-148b/152 impact the regulation of MHC class I (MHC-I) molecules in TLR4-triggered inflammatory responses. **(A)** The expression of MHC-I was detected in lipopolysaccharide (LPS)-stimulated bovine endometrial epithelial cells (bEECs). **(B)** ELISA analysis of IL-1β and TNF-α in bEECs transfected with bta-miR-148b/152 mimics and then stimulated for 4 h with LPS. **(C)** ELISA analysis of IL-1β and TNF-α in bEECs after ligation with control antibody IgG or monoclonal antibodies to MHC-I and stimulation for 4 h with phosphate-buffered solution or LPS. Data are presented as the mean ± SEM of three independent experiments. *P < 0.05 compared to the negative control.

Although some studies on MHC-I have shown it alleviates TLR-triggered innate inflammatory responses (23), no research has demonstrated the effects of miRNA-mediated MHC-I molecules on TLR4-induced immune responses in bEECs. Thus, to detect whether miRNA-mediated downregulation of MHC-I heavy chain expression impacts TLR4-induced inflammatory responses, LPS (a TLR4 ligand) was administered to MHC-I heavy chain-silenced bEECs. bta-miR-148b/152 mimics were used to inhibit MHC-I heavy chain expression. First, MHC-I levels were detected in cells treated or not with LPS using a western blot method. The results showed that MHC-I expression was relatively increased by LPS challenge (Figure 4A). Then, the effects of silencing the MHC-I heavy chain on cytokines was investigated by ELISA in LPS-stimulated bEECs. The results showed that MHC-I heavy chain silencing increased the secretion of the pro-inflammatory mediators IL-1β and TNF-α in response to LPS challenge compared with control mimics (Figure 4B). Moreover, cross-linking of MHC-I molecules on bEECs showed that cells crosslinked with antibodies to MHC-I (anti-HLA class I) produced lower amounts of pro-inflammatory cytokines (TNF-α, IL-1β) than did cells treated with control antibodies (Figure 4C). These data indicated that silencing of the MHC-I heavy chain by bta-miR-148b/152 enhanced LPS-induced inflammatory responses in bEECs.

### bta-miR-148b/152 Impacts the Regulation of MHC-I Molecules on TLR4 and Its Downstream Pathway Activation

**Figure 5:**
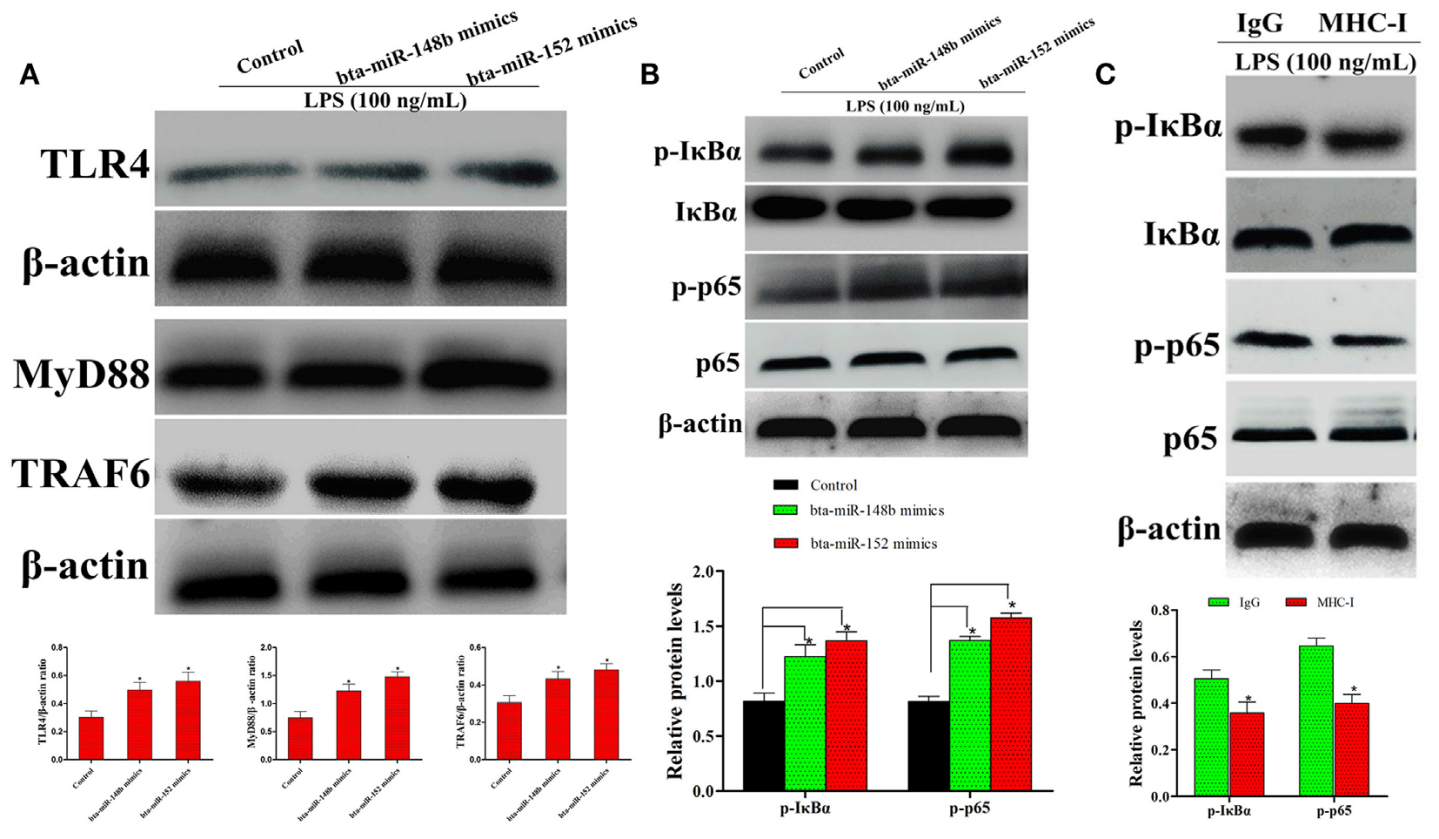
bta-miR-148b/152 impact the regulation of MHC class I (MHC-I) molecules on TLR4 and its downstream pathway. **(A)** The expression of TLR4 and its downstream factors myeloid differentiation factor 88 (MyD88), TRAF6 were determined by western blot in MHC-I heavy chain-silenced bovine endometrial epithelial cells. **(B)** The expression of IκBα and p65 protein was determined by western blotting. Phosphorylated IκBα and p65 were analyzed using phospho-specific antibodies. **(C)** The effect of MHC-I ligation on nuclear transcription factor (NF)-κB activation was further investigated through immunoblot assay. β-actin was used as an internal control. Data are presented as the mean ± SEM of three independent experiments. *P < 0.05 compared to the negative control.

It is well known that LPS is a TLR4 agonist. As shown by western blot assay, compared to the control group, MHC-I heavy chain-silenced bEECs showed increased TLR4 expression after LPS stimulation. MyD88 and TRAF6 are the TLR4 signaling downstream molecules. Therefore, we also investigated the expression of these adaptor molecules in MHC-I heavy chain-silenced bEECs through western blotting. The results showed that the expression of MyD88 and TRAF6 was increased in MHC-I heavy chain-silenced bEECs (Figure 5A).

The NF-κB pathway, which is downstream of TLR4 signaling, plays an important role in regulating the expression of inflammatory cytokines (25). To explore whether MHC-I heavy chain silencing intersects with theTLR4 signaling pathway in bEECs, the NF-κB pathway was analyzed by western blotting. The activation of NF-κB (IκBα and p65) was enhanced in LPS-stimulated MHC-I heavy chain-silenced bEECs (Figure 5B). As MHC-I ligation alleviates TLR4-induced inflammatory responses, the effects of MHC-I ligation on the TLR4 pathway was further investigated. The results showed that cross-linking MHC-I molecules on bEECs contributed to the reduction in NF-κB pathway activation in response to LPS challenge (Figure 5C). The above data demonstrated that bta-miR-148b/152 could enhance LPS-triggered inflammatory responses by targeting the bovine MHC-I heavy chain.

### MHC-I Heavy Chain Knockdown Increases the LPS-Induced Inflammatory Response in BEND Cells

**Figure 6:**
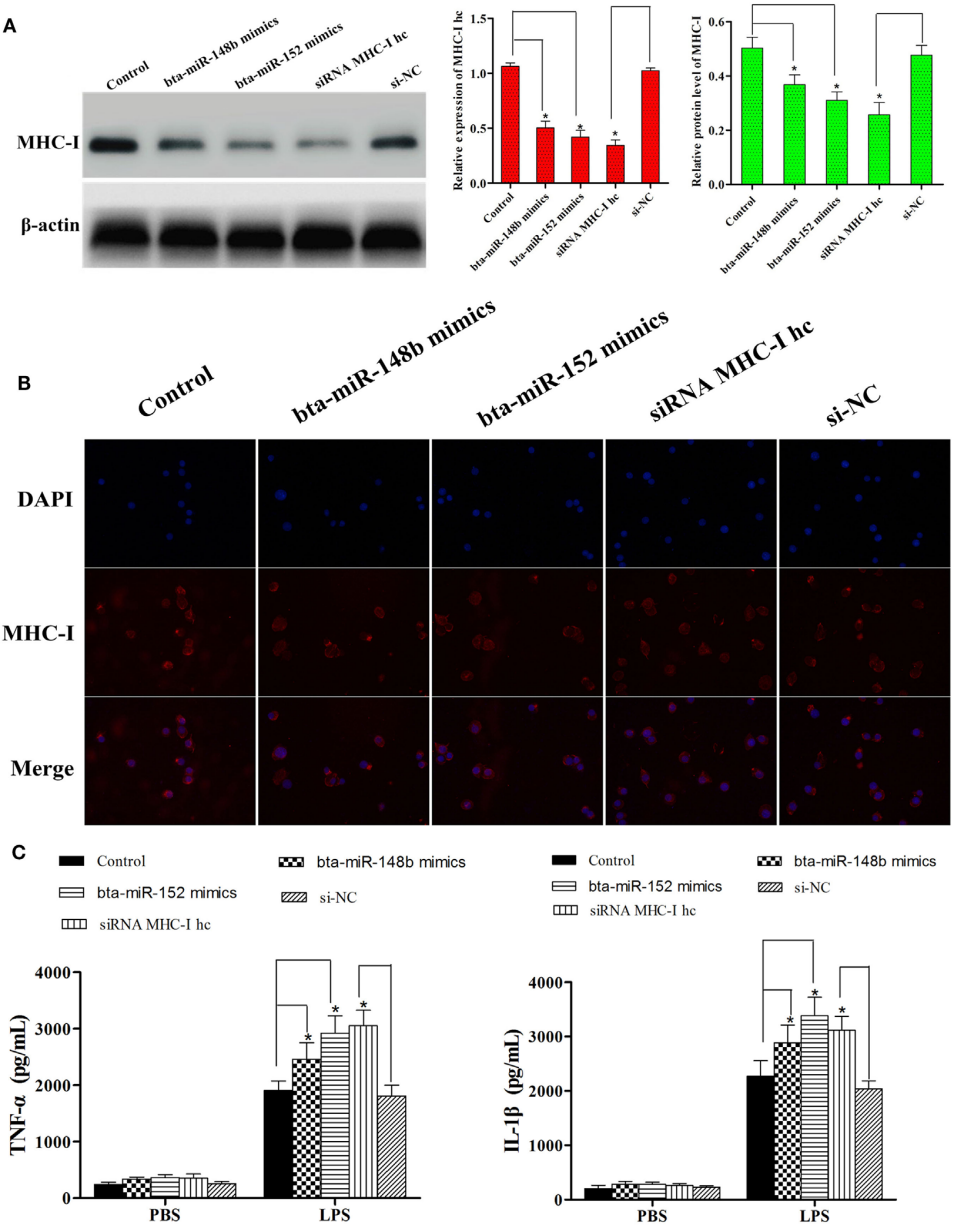
MHC class I (MHC-I) heavy chain knockdown increases lipopolysaccharide-induced inflammatory response in BEND cells. **(A)** The cell line was transfected with bovine MHC-I heavy chain small interfering RNA (siRNA), bta-miR-148b/152 mimics, or control siRNA; then, the expression of MHC-I heavy chain was detected by qRT-PCR and Western blot assays. β-actin was used as an internal control. **(B)** The expression of MHC-I heavy chain was further determined by immunofluorescence method. **(C)** The cell line was transfected with bovine MHC-I heavy chain siRNA, bta-miR-148b/152 mimics, or control siRNA; then, the expression of IL-1β and TNF-α was determined by ELISA in MHC-I heavy chain-silenced bovine endometrial epithelial cells. Data are presented as the mean ± SEM of three independent experiments. *P < 0.05 compared to the negative control.

We further investigated the effects of knocking down the MHC-I heavy chain on the LPS-stimulated inflammatory process in the BEND. siRNA specific to the bovine MHC-I heavy chain and bta-miR-148b/152 mimics both markedly decreased the expression of the MHC-I heavy chain (Figures 6A,B). The results indicated that the MHC-I heavy chain was silenced by specific siRNA and bta-miR-148b/152 mimics.

After validating MHC-I heavy chain silencing, secretion of the pro-inflammatory cytokines IL-1β and TNF-α was determined with the ELISA method. As shown in Figure 6C, knockdown of the MHC-I heavy chain increased the production of TNF-α and IL-1β, compared with control siRNA or control mimics treatment.

### MHC-I Heavy Chain Knockdown Enhanced the Expression of TLR4 and Activated the NF-κB Pathway in BEND Cells

**Figure 7:**
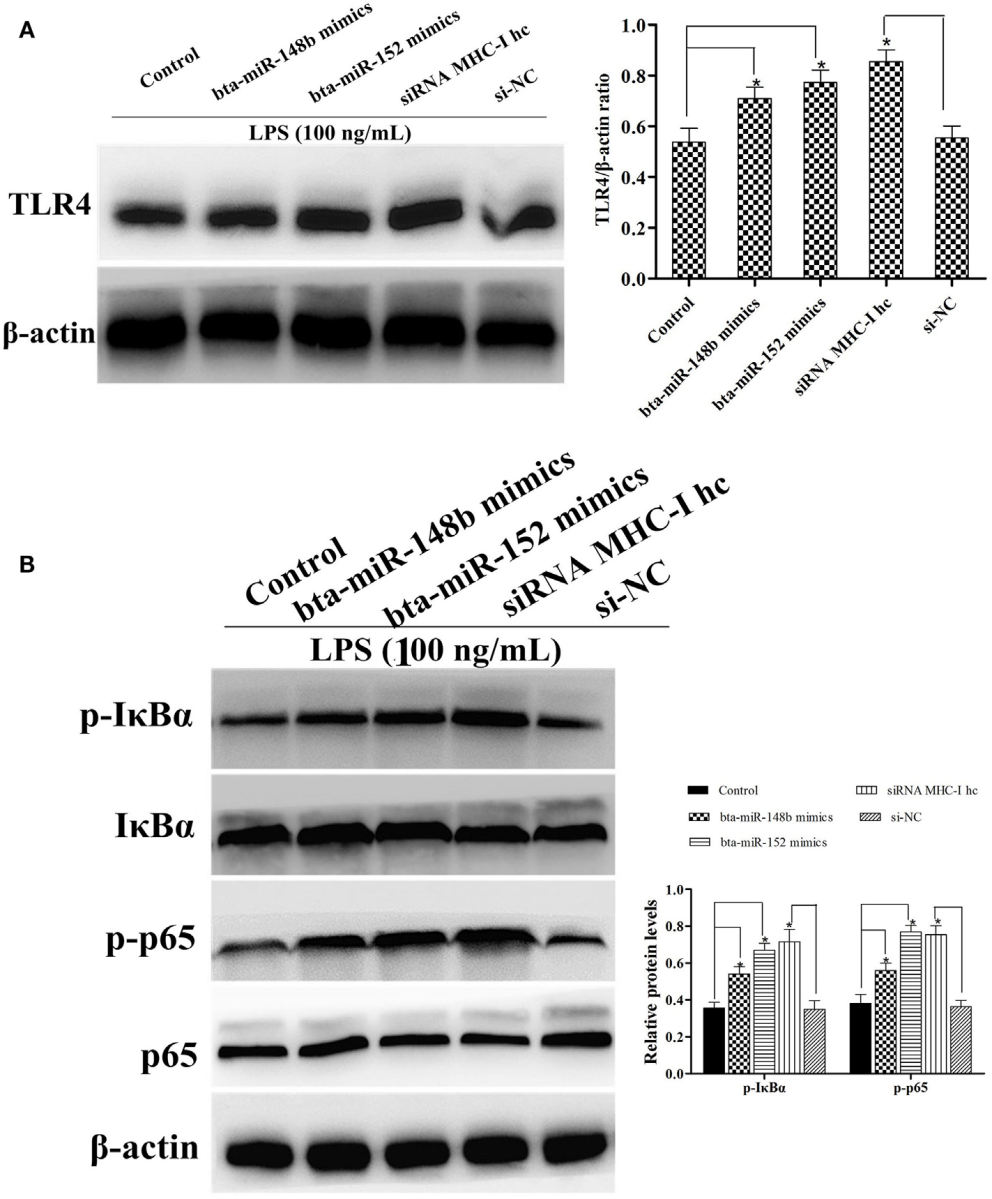
The expression of TLR4 and its mediated signaling pathway in MHC class I (MHC-I) heavy chain knockdown BEND cells. **(A)** TLR4 expression level was determined by western blot assay in BEND cells. **(B)** The expression levels of IκBα and p65 protein were detected by a western blot assay in lipopolysaccharide (LPS)-stimulated BEND cells exposed to specific small interfering RNAs (siRNAs), bta-miR-148b/152 mimics, or control vectors. Phosphorylated IκBα and p65 were analyzed using phospho-specific antibodies. β-actin was used as an internal control. Data are presented as the mean ± SEM of three independent experiments. *P < 0.05 compared to the negative control.

To further corroborate the result that LPS stimulation increased the expression ofTLR4 and NF-κB pathway activation in MHC-I heavy chain-silenced bEECs, we explored MHC-I molecule function during LPS stimulation of BEND cells using western blotting. BEND cells in which the gene encoding the MHC-I heavy chain was silenced by specific siRNA or those treated with bta-miR-148b/152 mimics showed a greater increase in TLR4 expression in response to LPS challenge than those transfected with control siRNA or control mimics (Figure 7A). The activation of the NF-κB pathway was determined in LPS-stimulated BEND cells with specific siRNA, bta-miR-148b/152 mimics, or the control vector through western blotting. As shown by western blot assay, the phosphorylation of IκBα and p65 protein levels were also increased by deleting the MHC-I heavy chain in BEND cells (Figure 7B).

### MHC-I Heavy Chain Knockdown Promoted TLR4 Signaling in BEND Cells

**Figure 8:**
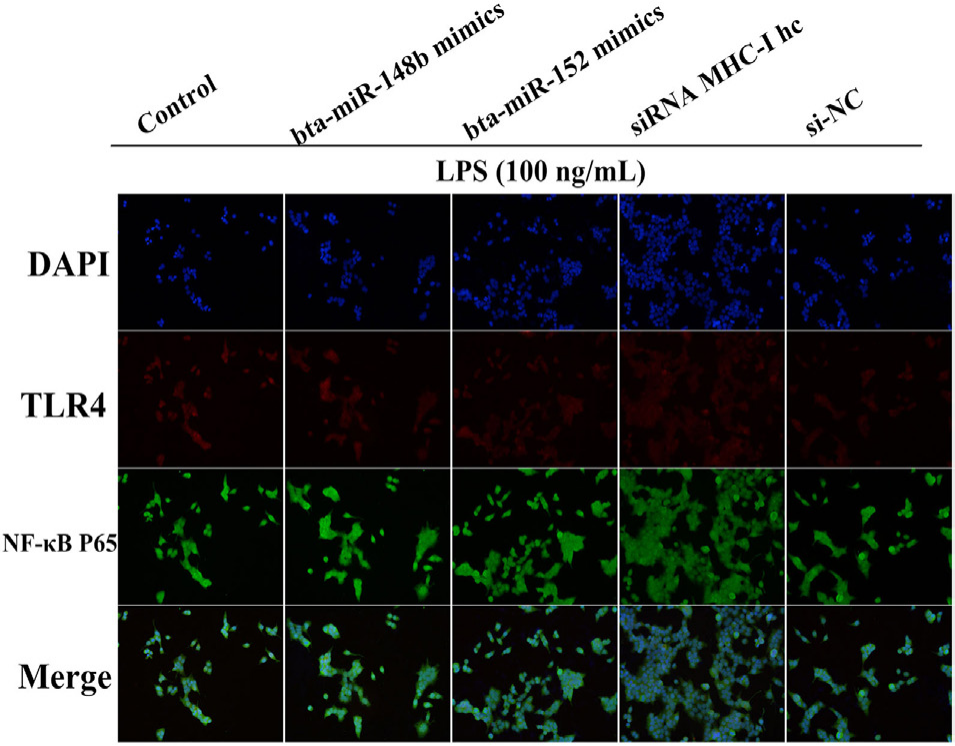
MHC class I (MHC-I) heavy chain knockdown promotes TLR4 and nuclear transcription factor (NF)-κB p65 expression in BEND cells. The expression of TLR4 or NF-κB p65was further confirmed by an immunofluorescence technique in lipopolysaccharide (LPS)-stimulated BEND cells. BEND cells were stimulated with LPS before TLR4 (red) or NF-κB p65 (green) expression was detected by immunostaining, and nuclei were stained with DAPI (blue).

Moreover, further studies were performed to determine TLR4 and NF-κB p65 expression in MHC-I heavy chain gene knockout BEND cells that had been challenged with LPS *via* an immunofluorescence assay. We found that TLR4 (red) and p65 (green) expression both increased upon deleting MHC-I heavy chain in BEND cells (Figure 8). The results suggested that bta-miR-148b/152 could promote TLR4-triggered inflammatory responses by targeting the bovine MHC-I heavy chain.

### MHC-I Suppressed TLR4 Pathway by Fps Signaling Activation

**Figure 9:**
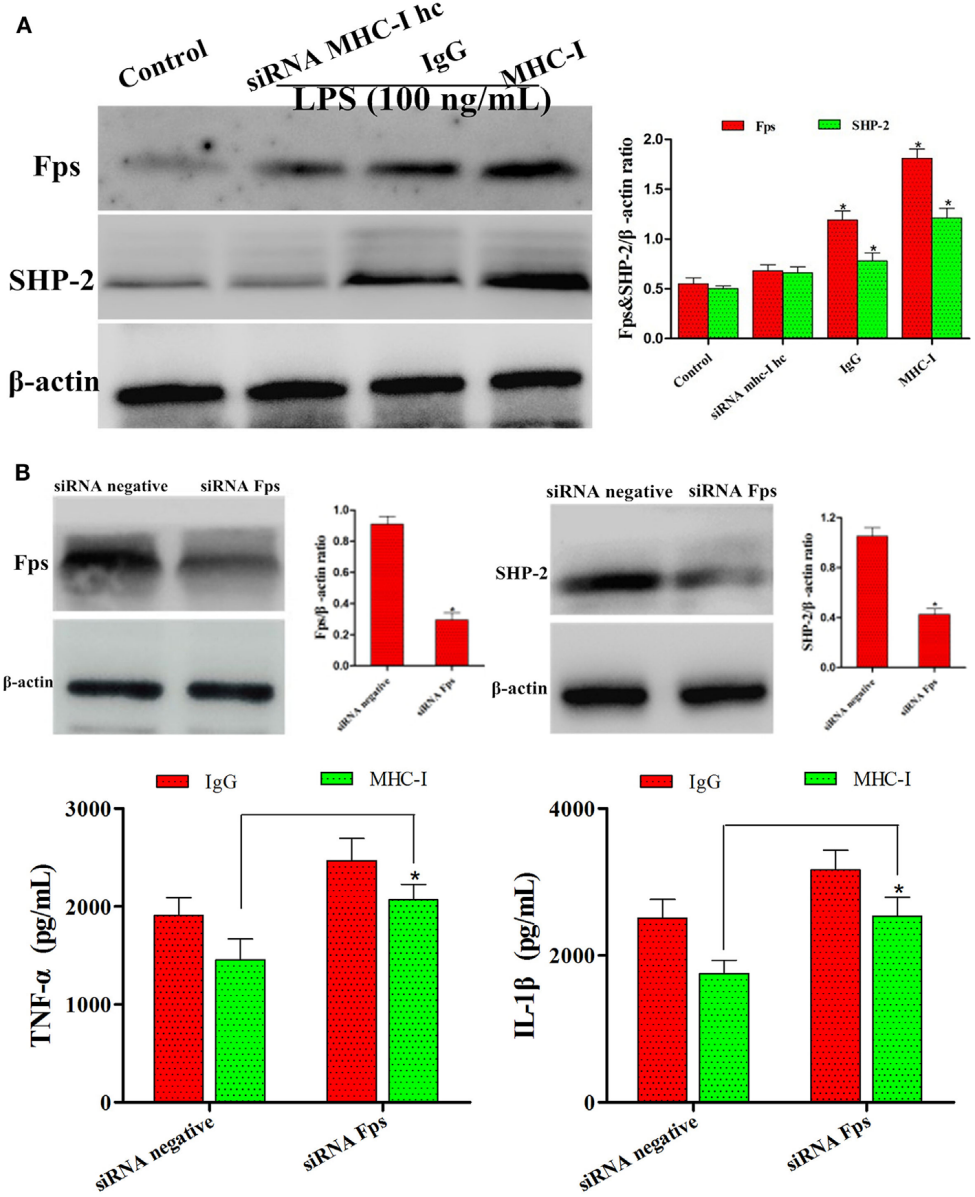
MHC class I (MHC-I) suppressed TLR4 pathway *via* Fps signaling activation. **(A)** The expression of Fps and SHP-2 were determined by immunoblot assay. **(B)** Immunoblot analysis of Fps and SHP-2 after transfected with control small interfering RNA (siRNA) or Fps siRNA, and then detected the inflammatory reactions by ELISA kits. β-actin was used as an internal control. Data are presented as the mean ± SEM of three independent experiments. *P < 0.05 compared to the negative control.

It has been reported that MHC-I molecule negatively regulated TLR-induced inflammatory reactions through the Fps-SHP-2 pathway (23). Thus, the potential mechanism of the inhibition of TLR4 pathway *via* MHC-I molecule was further investigated. As displayed in Figure 9A, the results displayed that MHC-I molecule crosslinkage enhanced its interaction with Fps. Besides, after stimulation with LPS, the SHP-2 phosphorylation was reduced in which the gene encoding Fps was interfered and then enhanced the pro-inflammatory cytokines secretions (Figure 9B). These above results indicated that, and the MHC-I molecule negatively regulated TLR4-induced inflammatory reactions may through enhancing the Fps-SHP-2 pathway activation.

## Discussion

IFN-τ is well known as a vital pregnancy cytokine and is secreted by trophoblast cells during implantation (26). It has been reported that many of the immune response genes are under the control of IFN-τ in bEECs (27). Evidence accumulated so far strongly suggests that IFN-τ plays a vital role in the early immunological interactions between the maternal–fetal interface, exerting regulatory effects of MHC molecules on cellular expression (2, 10). In this experiment, we observed that IFN-τ promoted MHC-I expression and then alleviated the inflammatory response in bEECs. miRNA, or non-coding small RNA, have become known as novel molecular regulators of numerous genes and pathways involved in immune responses (28). Several studies have demonstrated that miR-148/152 is associated with the immune process (15, 29, 30). Thus, we believe that IFN-τ regulates MHC-I molecule expression and function, possibly by affecting expression of some specific miRNAs. In the present study, we observed that bta-miR-148b/152 were inversely correlated with the MHC-I heavy chain in IFN-τ-stimulated bEECs.

MHC class I molecules play a vital role in the immune system, and the high diversity of these genes makes it possible for populations to survive in the presence of rapidly developing pathogens (31). It has been found that MHC-I class molecule (BoLA-I) antigens are associated with the occurrence of clinical mastitis (32, 33). Moreover, TLR is the main regulator of the initiation of the innate immune response, which perceives microorganism infection and triggers various antibacterial and inflammatory reactions (34). A previous study showed that MHC-I molecules are engaged in cross talk with TLR-triggered inflammatory responses (23). In addition, Liu et al. also showed that miR-148/152 can inhibit TLR-triggered MHC-II expression, which may act as a fine-tuner in the regulation of immune responses (14). Accordingly, we hypothesized that bta-miR-148b/152 might be involved in the regulation of MHC-I and TLR receptors.

TLR4, as a major TLR receptor, specifically identifies LPS, and its activation initiates intracellular signaling, including the MyD88/NF-κB pathway (35). The activation of NF-κB leads to secretion of the pro-inflammatory mediators TNF-α and IL-1β (36). IL-1β and TNF-α are usually considered pro-inflammatory mediators and have important roles in the immunologic system (37). In the current research, we found that knockdown of the MHC-I heavy chain increased the expression of the pro-inflammatory mediators TNF-α and IL-1β in LPS-stimulated bEECs. These results indicated that MHC-I deficiency increased TLR4-induced inflammatory reactions in bEECs.

Nuclear transcription factor-κB controls a broad spectrum of target gene expression, as a key coordinator of inflammatory and immune responses, as well as cell differentiation, proliferation, apoptosis, and survival (38, 39). The present study showed that NF-κB activation was enhanced in LPS-stimulated, MHC-I heavy chain-silenced bEECs. Moreover, Fps, a non-receptor tyrosine kinase, has vital roles in the process of inflammation and immunity (40). SHP-2 as a downstream factor of Fps and its expression pattern is similar to that of MHC-I molecules (23, 41). The present results suggested that Fps associated with SHP-2 and that this association inhibited the TLR4 responses by MHC-I molecule. Our data provide evidence that the MHC-I molecule negatively regulated TLR4-induced inflammatory reactions may through the Fps-SHP-2 pathway.

**Figure 10:**
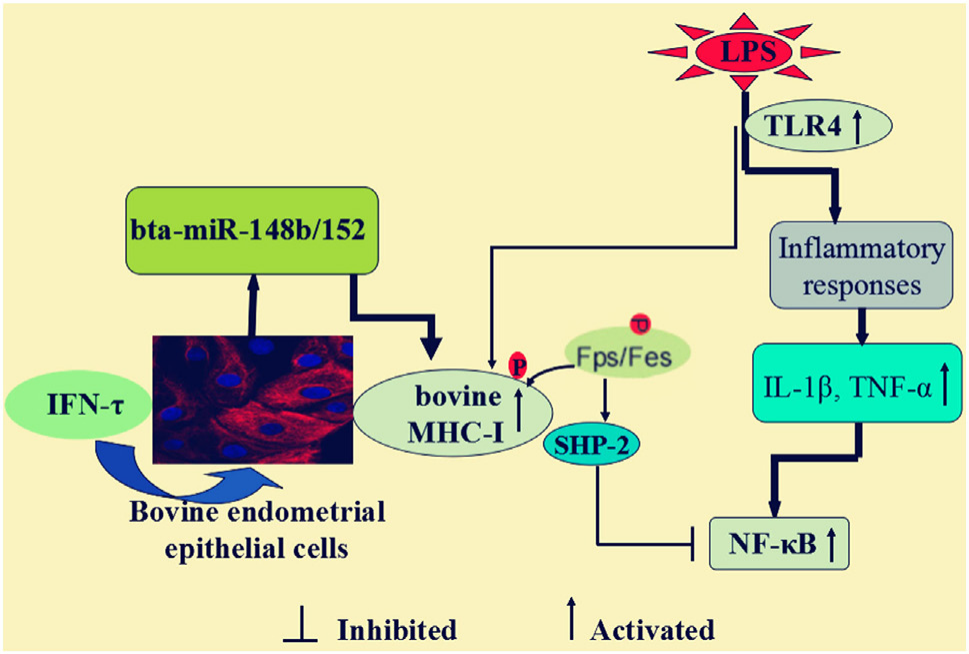
Scheme summarizing the IFN-τ promoted the MHC class I (MHC-I) expression, and then alleviated the inflammatory response in lipopolysaccharide (LPS)-induced bovine endometrial epithelial cells. The mechanism was that bta-miR-148b/152 could promote TLR4-triggered inflammatory responses by targeting bovine MHC-I heavy chain, and the MHC-I molecule negatively regulated TLR4-induced inflammatory reactions may through the Fps-SHP-2 pathway.

In conclusion, as illustrated in Figure 10, our data demonstrated that IFN-τ promoted MHC-I expression and then alleviated the inflammatory response in bEECs, and the mechanism is through affecting the expression of bta-miR-148b/152. Furthermore, we also found that bta-miR-148b/152 could promote TLR4-triggered inflammatory responses by targeting the bovine MHC-I heavy chain, and the MHC-I molecule negatively regulated TLR4-induced inflammatory reactions may through the Fps-SHP-2 pathway. Our discovery offers novel insight into the negative regulation of the TLR4 signaling pathway and elucidates the mechanism by which bovine MHC-I molecules control congenital inflammatory reactions.

## Author Contributions

HW and GD contributed to the conception and design of the study. HW, KJ, and SG performed experiments, collected samples, and accomplished the laboratory investigations. JY and GZ acquired data. HW, KJ, and GD conducted the data analysis. HW and KJ drafted the manuscript. HW, CQ, and GD modified the manuscript. All the authors have read and agreed to the final manuscript.

## Conflict of Interest Statement

The authors declare that the research was conducted in the absence of any commercial or financial relationships that could be construed as a potential conflict of interest.
